# Supplementary figures and images for: Valorization of Honduran Agro-Food Waste to Produce Bioplastics
Source: Polymers (Basel). 2023 Jun 9;15(12):2625. doi: 10.3390/polym15122625 (PMC10305083; doi:10.3390/polym15122625)

## Supplementary material

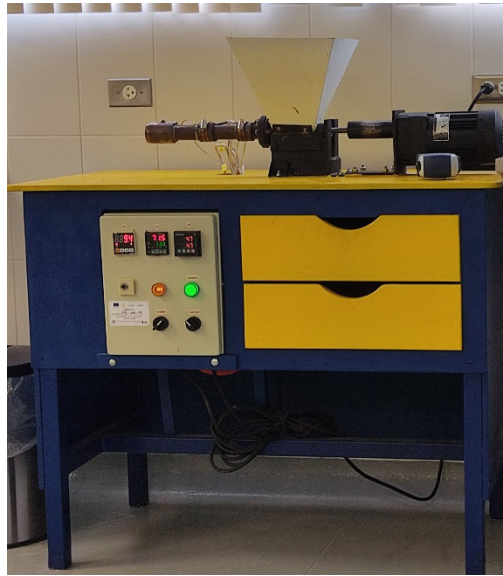

**Figure S1.** In-house manufactured extruder.

Supplement: Supplementary file 1 [file polymers-15-02625-s001.zip › polymers-2401536-supplementary.pdf]
